# Perspectives on the Teaching and Learning of Non-Verbal Communication Skills: Tutor, Simulated Patient and Student Experiences of a Rapid Transition to Online Learning

**DOI:** 10.1007/s40670-026-02666-y

**Published:** 2026-02-13

**Authors:** Riemke Aggio-Bruce, Georgia Pike-Rowney, Amy Campbell, Soha Rizvi, Alexandra L. Webb, Krisztina Valter, Lillian Smyth

**Affiliations:** 1 School of Medicine and Psychology, College of Science and Medicine, Australian National University, Florey Building 54, Canberra, Australian Capital Territory 2601 Australia; 2 Centre for Classical Studies, School of Literature, Languages and Linguistics, College of Arts and Social Sciences, Australian National University, Canberra, Australian Capital Territory Australia

**Keywords:** Clinical skills, Communication skills, Medical education, Online learning

## Abstract

**Purpose:**

While nonverbal communication skills (NVCS) are an important contributor to building a clinical alliance, they are often included implicitly rather than explicitly in medical education programs. Ambiguity around if (and how) NVCS are *taught, learned or developed* limits the flexibility of implementation of NVCS education in medical programs. This gap in understanding is further highlighted by recent pushes toward online learning environments. This study aimed to identify the perceptions of clinical skills tutors, simulated patients, and students around learning NVCS and how that translates to an online context.

**Methods:**

A qualitative study was conducted using a series of semi-structured interviews with clinical skills tutors, simulated patients and medical students from an Australian medical program. The resulting data was analyzed using reflective thematic analysis.

**Results:**

We classified participant construals of how NVCS are taught and learned into four themes: ‘explicit teaching’, ‘experiential learning’, ‘observational learning’ and the importance of ‘feedback’. In the transition to online learning, the process of learning NVCS through explicit teaching was largely thought to be retained. Experiential and observational learning were retained in limited forms and some elements of feedback were lost.

**Conclusions:**

This study has identified new perspectives of stakeholders in teaching and learning NVCS online for use in clinical contexts. Learning NVCS online can be beneficial in preparing students for telehealth consultations. However, a better understanding of how skills used online and in face-to-face environments correspond is required to guide the development of innovative and engaging methods for the teaching and learning of NVCS.

**Supplementary Information:**

The online version contains supplementary material available at 10.1007/s40670-026-02666-y.

## Introduction

Non-verbal communications, including eye-contact, positioning, physical touch and facial expressions, and the ability to convey and recognize these behaviors [1], are critical to the clinical interaction. A meta-analysis by Henry et al. [2] indicated that a greater degree of physician eye contact, facial expressiveness, direct body language, and closer proximity are all associated with greater patient satisfaction and comprehension of clinical instructions [2]. Additionally, a study of non-verbal clinical skills (NVCS) in final year medical students during history taking demonstrated a correlation between non-verbal communication and relational empathy [3]. Further, in role-play scenarios, simulated patients were rated by trainers as less distressed, more engaged, and more dominant in the consultation when interacting with medical students who scored more highly on measures of non-verbal sensitivity. These same students were also more liked by the simulated patients [4]. Conversely, clinician *dis*engagement with these skills may be associated with malpractice [5]. As such, NVCS should form a core component of training in the health professions and be prioritized and protected in curriculum changes and transitions. This need to prioritize NVCS teaching and learning was thrown into sharp relief following the rapid transition to online teaching and learning following the COVID-19 outbreak. Attempting to teach clinical skills online, with limited visual cues and no physical proximity, made clear the importance of these non-verbal elements of the clinical interaction.

The current study considers the implementation of teaching and learning of NVCS in the context of medical training in Australia, but the principles equally apply to other health professions in other contexts. In the Australian context specifically, the ability to demonstrate NVCS appropriate to a clinical setting, including ‘capacity to recognize, interpret and respond appropriately to non-verbal cues’, and ‘consistent and appropriate awareness of own non-verbal behaviors’ is one of the inherent requirements for medical training, as outlined by the Australian and New Zealand Association of Medical Deans [6]. However, this is often assessed, rather than taught, and it is rare for a medical program, locally or internationally, to have a specific curriculum for explicit teaching and learning of non-verbal skills for clinical settings [7]. This lack of explicit teaching consigns these skills to the “hidden curriculum” [8], navigation of which can lead to feelings of hypocrisy and cynicism in students. It can also disadvantage some students, as the skills for navigating the gap between the explicit and hidden curricula can be tied to social class, ethnic and racial background [8]. As such, the teaching and learning of these skills remains an open question internationally and the current study uses the case study of one Australian medical school to provide preliminary evidence to inform further conversation.

NVCS relevant to the medical setting are commonly taught implicitly, with a focus on creating an environment that supports learning of these skills [9]. In the pre-clinical years of medical training (i.e. where students are engaged in classroom teaching, rather than on clinical rotations), NVCS are developed through learning vectors that include role-play and modelling, reflection, feedback, and assessment [7, 10]. These learning vectors have been shown to promote experiential learning of NVCS, and to increase clinicians’ awareness and perceptions of these skills [11, 12]. As students move into the clinical phase, an apprenticeship model is often adopted, whereby students learn ‘on the job’ from more experienced clinicians [9].

This broad-spectrum approach to education, wherein the environment contains multiple vectors for learning, is excellent pedagogical practice. However, it also means it is difficult to disentangle, conceptually, the benefits of individual learning methods and thus introduces a problem during curriculum transformation. Without a precise understanding of the roles that the components of the teaching and learning play in the development of NVCS, we don't know what must be preserved and what we can lose when making curricular changes in a particular context. This is a more general problem, in terms of know what the mechanism of effect is. It is also, however a specific practical problem when unexpected external forces necessitate curriculum change.

### NVCS Teaching and Learning Online

As an example of this process, the sudden requirement for physical distancing in Australia brought on by the COVID-19 pandemic led to a shift of both educational and medical consultations to telecommunications platforms (e.g. use of video-conferencing software). In some instances, known pre-existing benefits to telehealth/mixed delivery allowed for quick adaption, however the transition highlighted the gap in understanding of how NVCS are taught for a clinical setting. In moving to the online teaching space, educators had limited understanding of how, for example, the loss of physical co-location, the limited visual field, and the interruption of opportunities for eye-contact and physical touch might impact on the learning of NVCS by medical students.

As part of this transition, some limitations to non-verbal communication were clear: technological limitations such as screen size, video quality and poor internet connection [13] made an immediate impact. Consequently, medical training shifted away from communication towards a focus on learning procedural skills, e.g. physical examination [14]. In some instances, however, students were still assessed on non-verbal communication in an online setting [15]. These challenges to training and assessment were heightened by a perceived loss of nonverbal information when moving education to an online context [16], and difficulties for students and examiners to pick up on empathetic cues in an online setting [13]. Recommendations for engaging in an online space have accordingly been developed in recent years, such as educating students on adapting eye-contact for video conferencing [17]. It remains unclear if explicit guidelines, such as these, are sufficient to adequately prepare students to provide quality healthcare in a flexible (online and in person) environment.

## The Current Study

While there is existing literature on models and processes of NVCS teaching and learning, the level of precision required for curriculum change, particularly in unprecedented circumstances, demands a richer understanding of the actual teaching and learning context. In order to understand the fine details of how these processes play out in the classroom, we examined the experiences and insights of the participants in that environment. Key informant interviews with tutors, simulated patients and medical students allowed us to build a picture of the ways in which the learning objectives, transmission of content, modelling, role-play and feedback were operationalized in the online classroom. The short-notice transition to an online context during the COVID-19 pandemic provided an excellent trigger for interview participants to reflect on both the experience of the online teaching but also, comparing to their previous teaching and learning experiences, what had been lost and/or preserved in the transition online. We anticipated that this interruption might facilitate greater insight into aspects of the process that might go un-detected during business-as-usual teaching. The key informant interviews were structured around two key questions: 1) what are the perceptions of tutors, simulated patients and students of how NVCS are developed during medical training (e.g. can they be taught?) and 2) how well these learning processes and experiences had translated to an online environment.

We aimed to capture a 360-degree perspective of key stakeholders (tutors, simulated patients, and students) involved in history taking sessions, to gain a broad understanding of shared and unique viewpoints. This data allowed a more nuanced understanding of the process from multiple perspectives and can inform the adaptations we make to healthcare professional education that has evolved post-COVID-19 pandemic into a hybrid delivery world.

## Methods

### Context

The study was conducted in a graduate-entry, 4-year medical program in a small city in Australia. The program is divided into two phases; the pre-clinical years (1 and 2), wherein students are primarily on a university campus and clinical skills are trained in formal tutorials with simulated patients and supervised patient contact, and the clinical years (3 and 4), in which students are embedded in clinical rotations and involved in patient care. The school is relatively small (~ 100 students per year cohort) and the medical program has a significant rural-practice-oriented component in the training, as well as a substantial research component. The local context in which the medical program and health system are embedded is a small city, with significant in- and out- migration flows associated with education and civil service. The majority of students in the program relocate to this city for the purposes of pursuing the medical degree.

### Design

This study used a qualitative interview design with stratified sampling of the three relevant populations. Participants were asked to discuss their experiences and perspectives retrospectively, after the phenomenon of interest (the first-semester clinical skills training) had occurred. The study was a rapid-response study conducted during the early stages of the COVID-19 pandemic, to explore the changes that arose through the natural experiment of forced online learning. Recruitment and data collection were done by the second author (who is neither a health professional nor health professions student).

### Participants

Participants in this study were affiliated with a small, 4-year graduate-entry medical degree at an Australian institution. The participants were recruited through advertisements on the institution’s digital learning management system, social media, and student/staff email lists. All preclinical medical students, clinical skills tutors and simulated patients who had engaged with the clinical skills program were invited to interview, however, participation was limited to those who had engaged with the program in 2019 and/or 2020 and were currently enrolled at the time of data collection (2020). In this content, “clinical skills tutors” refers to a particular role in the medical teaching curriculum wherein clinicians (active or retired) facilitate small-group tutorials of students to practice skills, such as history taking. “Simulated patients” in this context refers to those who are trained to “play” the role of the patient to simulate specific presentations in these clinical skills tutorials. The recruitment of simulated patients was a priority, as these perspectives are only rarely included in the literature [18]. A focus on students, on the other hand, is comparatively common (for a recent example, see Dewi et al. [19]) Participation in the research was voluntary for all stakeholder groups and no incentives were offered for involvement.

We intended to have a stratified sample with at least 4 participants from each stakeholder group (i.e. 4 tutors, 4 simulated patients and 4 students). However, due to the voluntary nature of the study, the strain the student sample were already under at the time, and the time-limited data collection window, our student sample comprised only 2 participants. As the medical school under examination is small (100 students per cohort), this represents about 10% of the available student sample and student data should be interpreted with caution. We did not refuse any participants, so additional tutors and simulated patients were interviewed.

In total 13 participants provided informed consent, including 5 clinical skills tutors, 6 simulated patients and 2 first-year medical students. All participants had been involved in the “history taking” block of clinical skills training in semester 1 of 2020 and data were collected after the conclusion of the full block of clinical skills training. Owing to the pandemic outbreak, this teaching was all in an online-only format.

We did not set any *a priori* expectations for the number of interviews required. As reviewed in Braun and Clarke [20], recommendations in the literature for minimum sample size to reach “saturation” range between 6 and 16. However, as also reviewed in that commentary [20] saturation is itself a problematic concept that does not align with a reflexive thematic analysis approach (RTA), which is the approach we have taken to our analysis. Saturation as a goal rewards coarse categorization in coding, and narrowly conceptualized themes. Themes identified through this process tend to function as “topic summaries”, rather than integrative understandings of patterns of shared meaning. While our final number of interviews would approximately meet the quasi-quantitative expectations for numbers of interviews found in the literature (for example, 12 per Guest and colleagues [21]), our sample size decisions were guided by ensuring representation of our target stakeholder groups, while also working with the reality of a small medical school and the difficult circumstances of the pandemic.

All participating tutors were retired from clinical work and had previously specialized in general practice. This was representative of the tutoring cohort as a whole. One tutor interview was excluded from analysis due to sharing of personally identifying information, that the authors felt might pose an unreasonable risk of re-identification. This transcript was considered thoroughly prior to exclusion and the analysis team determined that richness of the dataset relating to the teaching and learning of NVCS would not be reduced with its exclusion.

### Research Instrument

To investigate stakeholders’ perspectives on the nature, teachability and learnability of NVCS, a semi-structured interview protocol was designed (Appendix A). Interview questions were guided by previous literature [3] on non-verbal communication skills in history-taking assessments of undergraduate medical students. The questions were further developed with reference to the relationship of these skills with perceptions of empathy using the ‘Consultation and Relational Empathy Measure’ [22]. The final protocol was validated through consultation with the institution’s clinical skills academic coordinators and was then consensualized by the research team.

Each interview was opened with introductory questions concerning the participants’ backgrounds, occupations, and clinical skills experience. Next, participants were asked a set of questions regarding non-verbal communication skills in clinical settings. These explored participants’ perceptions of these skills, including specific examples of NVCS, and the role and impact they have on the clinical interaction. To conclude, participants were encouraged to reflect on the pedagogy of teaching and modelling NVCS, and the impact of transitioning to online learning during COVID-19. The interview questions were adapted as needed to suit emerging data.

### Procedure

The interviews were conducted in Australia throughout 2020, using online ‘Zoom’ video conferencing software [23]. The interviews were conducted via Zoom, as mandatory social distancing protocols were still in place in Australia at this time. Confidentiality was ensured through the use of unique meeting ID codes and password protection. Each interview was conducted as a one-on-one session with the second author, who has an interdisciplinary research background in music, health, classical studies and education. A small number of study participants were previously acquainted with this author due to belonging to the performing arts community in a small city, however, most participants were unfamiliar.

At the start of the interview, participants were given an information sheet providing context for the study, and oral consent was obtained. After addressing the interview questions in the protocol, participants were given additional time to provide further thoughts and feedback. Participants were also encouraged to contact the interviewer later if they wished to provide additional insights and were offered a copy of their transcript for amendment. One participant made minor grammatical corrections to their transcript. The duration of each interview varied, with a maximum length of one hour. The audio was recorded using an external digital recording device to ensure identifying metadata that could be captured by the video software was not stored with the audio recording.

### Data Transcription and Analysis

Following de-identification, the interviews were transcribed by four project investigators using automated transcription with human review and editing. The resulting data was then qualitatively analyzed by the four authors using a thematic analysis approach informed by Braun and Clarke’s Reflexive Thematic Analysis approach [24]. Analysis proceeded broadly in six phases: familiarization, coding, generating initial themes, developing and reviewing themes, refining and naming themes and writing up. The first, third and fourth authors engaged in phases 1 to 3 independently and collaborated with one another and the remaining authors to develop, refine, name and organize the themes (phases 4–6) for presentation. These three authors first familiarized themselves with the data by listening to and reading the transcripts, part of which was previously required for transcription. During this phase, key ideas and quotes from the transcripts were recorded. Codes were then generated using an inductive approach, due to the exploratory nature of the study. All codes were recorded in a codebook with definitions and relevant quotes. The resulting coding scheme was then consensualized and refined within the full research team. Following completion of the coding process, themes were identified and developed by clustering relevant codes together and considering relationships between them. The themes were then reviewed and refined in consultation with the full authorship team, and utilized in writing up the current paper.

### Positionality

All authors are involved, to a greater or lesser degree, with medical student training. Two authors were later-year medical students in the program at the time of their contributions. Note that the student authors were from the 2021-commencing cohort and worked with anonymized transcripts. The majority (4 of 5) remaining authors are continuing teaching staff associated with the medical program. As such, the research questions, methods and analysis will not be without the influence of our subjectivity, in terms of what works, what doesn’t and what’s important in medical education. In order to minimize the intrusion of our own values onto data collection, the development of the interview protocol and collection of the interview data were led by the second author- who has no experience teaching in the medical program. This reduced the effects of both interviewer expectations on the interactive data collection process, but also mitigated the potential for existing relationships to color data collection (as the second author was an “outsider” asking questions, rather than a known colleague).

The analysis process involved the full authorship team and, as such, may well have been colored by our own values as medical educators and students. Crucially, none of the authors are involved in the teaching of clinical skills in the medical program or contribute to the clinical history-taking training.

Finally, the interdisciplinary authorship team includes researchers from a broad range of backgrounds, but we acknowledge that we commenced the research from a point of consensus that NVCS were critically important and needed to be preserved and protected as teaching moved online.

## Results

We aimed to investigate two core questions with our interview protocol; 1) what are the perceptions of tutors, students and simulated patients of how NVCS are developed during medical training and 2) how these learning processes and experiences had translated to an online environment. As our intent was to describe a multi-perspective view of the same history-taking training experience, interview transcripts from the three participant groups (tutors, simulated patients, and students) were treated as one interconnected group. We identified four themes in participant understandings of the ways in which NVCS are taught and learned. These were: ‘explicit teaching’, ‘experiential learning’, ‘observational learning’ and the importance of ‘feedback’. Themes are described in detail below and example quotations are presented in Table 1.

**Table 1:** Key themes in understandings of how nonverbal communications skills are learned in medical training

Through explicit teaching	Experiential learning	Observational learning	Feedback
*“I don't know how useful it would be, for example to have a lecture on these things. Because I don't know if it's something that you can explicitly be taught*.” -Student 1	“*I think you learn from dealing with people over a period of time*.”- Simulated Patient 1	*“These things that you do you pick up from your tutors and you pick up from other people. I guess ways of empathizing with the patient”*- Student 1	*“Sometimes I might say, I wasn’t feeling that you were having good eye contact, sometimes I think you might have jumped in a bit too early”* – Simulated patient 4
*“Sometimes we’ll talk [directly] about having the desk between you and the patient, how’d that feel?”* –Tutor 2	*“I think role play is the best… given that it's nonverbal I think the face-to-face experience and then getting reactions.”-* Simulated Patient 2	*“We tend to develop it over time, and what one student did last week that worked really well you then see the next student doing the next week”-* Tutor 2	*“there were no comments made to me in the feedback saying ummm, your nonverbal manner needs work”* – Student 1
*“I get them to think about […] manner of voice, how easily they respond, hesitancy, eye contact, nervous ticks, twitches, fiddling…”* Tutor 5	*“I guess I’ve just sort of like picked up on it, […] maybe it should be, um, outlined a little bit more, because I think some people can struggle with that”-* Student 2	“*it's difficult to describe non-verbal skills in words, but if you see them modelled it can be like easier to compare and, do it that way*”- Student 2	*“they take it in turns, taking the history with feedback from their peers and feedback from the tutors at the same time […] it does work well”—*Tutor 4

In considering the transition to online learning, we used the four themes (Table 2) derived from participant responses in addressing question 1, as an organizing framework for the barriers and opportunities arising from the move online.

**Table 2:** Key themes in understandings of how nonverbal communications skills are learned when medical training moves online

Challenges to experiential learning	Challenges to observation	Challenges to feedback	Practical limitations	Opportunities
*“But basically [practicing on] Zoom becomes all verbal in a way because of Internet connection… You can’t actually put your hand on [the simulated patient’s] knee, or move closer to them, if you move closer to them it’s actually quite threatening in the Zoom situation.*”- Tutor 2	*“You can still see facial expressions and things like that, like if the simulated patient wants to act a bit annoyed or something like that, I think they can still get that across. Maybe a little bit less so things like posture […] if you're only looking at someone from the shoulders down.“* - Student 2	*“the feedback was focused on the structure of the interview, and I think that's because it's hard to see the whole body and hard to judge, for example hand gestures on zoom when they're out of the picture.”*- Student 1	*“But it’s been difficult to teach the nonverbal cues, the nonverbal parts of the exam, of the history taking, because a lot of the simulated patients have very poor Internet connection, so therefore we’ll often just get a frozen face and then talking.”-* Tutor 4	*“I was thinking about head titling and head nodding and thinking I do that all the time. The only reason that I know I do that all the time is because I can see myself on zoom”—*Tutor 4
*“[in person,]…they’ll have a discussion amongst themselves when they’ve gotten themselves a little bit lost… That doesn’t seem to be happening as much with the online stuff”*- Simulated patient 5	*“nonverbal stuff is, you're only seeing, you know, the patient’s head, which means a lot of the hand gestures and things are lost. Um, so it's not as good.”-* Tutor 3	*“it’s very hard to reinforce those nonverbal things”-*Tutor 2	*“I don't think it's made it by any means impossible to use non-verbal skills to your own benefit or to read the patient. I think it's just been different.”-* Student 2	*“this is the new norm [for medical practice], so we're going to be doing a lot more of this sort of stuff, so they need to have some training”-* Simulated patient 4
*“if I'm looking at you in the eyes. I'm looking down not looking at the camera. And so you've always, you've gotta learn to actually look into the camera, not at the image of the person.*”- Simulated patient 1	*“You miss some of the non-verbals because you’re only seeing this part [indicating top part of body].*”- Simulated Patient 6		*“but when it went to online, it was very much as long as you're looking at the camera, it's fine…“*- Student 1	

### Theme 1: Teaching NVCS Explicitly

Given the amount of literature indicating that NVCS are often taught using an implicit or apprenticeship model, it is notable that a theme identified in our participant responses was explicit, didactic teaching of NVCS. What we refer to as “explicit” teaching includes approaches such as learning through direct instruction, lecturing, written material and/or structured conversation. Tutors described how they explicitly verbally communicate with students around important aspects of NVCS, such as body positioning relative to the patient, eye contact and body language, and further discuss these aspects in the group-based teaching environment. In contrast, however, one student reflected that it would be difficult to create resources to explicitly teach NVCS, mainly due to patient individuality, stating that *‘…I'm not sure whether that actually belongs in the […] guide because as I was saying, every patient is different. I don't know if it would be so easy to create like a universal like this is how you need to communicate’ –* Student 1.

### Theme 2: Experiential Learning of NVCS

The second mechanism of learning we identified in our participant responses was experiential learning. In this process, the student learns by doing and is typically "taught" through exposure to contexts, people and tasks. Participants described both formal exposure (e.g. classroom role-play tasks) as well as exposure that might arise informally through life experience. The model of learning implied was one of first-person experience and reflection and was described in terms of "*doing*", "*trying*", "*getting reactions*", "*dealing with people*" and "*picking up on it*".

### Theme 3: Observational Learning

In learning through observation, students typically view and memorize the nonverbal behaviors of all three groups: tutors, simulated patients, and students, during the simulated patient interviews. These observations can be interpreted, responded to, and/or mimicked. For some, observing modelled nonverbal communication overcomes the limitations of verbal or written descriptions of effective NVCS, and how to use them appropriately.

### Theme 4: Feedback

We identified comments from all stakeholder groups that described feedback, through evaluation of strengths and weaknesses, as an important contributor to student development of clinical skills. We identified among the clinical skills tutors a multi-pronged feedback approach that highlights the utility of this shared experience training model. First, participants highlighted the importance of giving both positive and negative feedback based on student performance. This feedback ties the nonverbal aspects of history taking into explicitly taught and assessed verbal competencies. Second, participants described inviting comment from all three groups; tutors, simulated patients, and students, to give a broad understanding to the student about their NVCS development. Finally, feedback is used by educators to help with self-reflection. Tutors drew attention to, and made students aware of, non-verbal behaviors by asking students to give themselves feedback.

### Transition to Online Learning

In considering what is lost and gained in the move to online learning of NVCS, we employed our four themes as an organizing framework. Example quotations are in Table 2. First, *explicit learning* was not mentioned at all in relation to the move to online learning. This is likely because explicit teaching using direct verbal communication and written material is not hampered by a move to online communication. With regard to *experiential learning*, these processes of learning were somewhat retained online. However, specific skills that were mentioned as effective for the patient interview in an online platform differed from those used previously in a face-to-face environment. Therefore, while experiential learning might still work in online training for an online environment, the skills learned online may not translate to a face-to-face interaction. Particularly, students had not had the opportunity to experience physical proximity or full-body language and learn from them. Additionally, the experiential process of learning through exposure to people was affected in the move online, where ongoing and incidental discussions among students (i.e. peer to peer) during class time and patient interviews did not occur as frequently or as smoothly online.

*Learning through observation* was perhaps the most affected by the online transition. Participants described limitations to the online model of learning through observation as centered around the restricted visual field - participants are only able to see one another from the shoulders and above. The ability to perceive body language from the full body is lost in this context. There is also the limit to simulating eye-contact, as the camera and the video of the interlocutor are not co-located on most devices. With this considered, participants reported that observational learning was retained online under certain conditions. So long as the picture was clear, and they were looking at the camera, many of the participants believed that facial expressions, for example, could still be picked up on. Further to this, using online video-based platforms created an opportunity to *see themselves* in a way not possible in person, enabling self-reflection to better understand their *own* non-verbal signals. With regard to *learning *via* feedback,* students felt that in the transition to online learning, the combined approach to feedback was lost, with a greater emphasis placed on verbal aspects.

Finally, participants reported a range of practical limitations of the online learning context. Participants from all three groups identified poor internet connection, and camera/equipment setup as primary limitations to non-verbal communication outside of the core four themes described above. Training in optimal technological setup was described as an opportunity to address these limitations and benefit telehealth training.

## Discussion

The current qualitative study explored the perspectives of tutors, simulated patients, and medical students on the teaching and learning of NVCS in medical education for in-person and online contexts. We identified four themes that underpin participants’ perspectives of learning NVCS: explicit teaching, experiential learning, observational learning, and feedback. These themes were also considered in understanding participants’ experiences of the move to online learning and teaching of NVCS, and what might be lost or gained in that transition.

### Findings: What are the Perceptions of Tutors, Simulated Patients and Students of How NVCS are Learned During Medical Training?

While explicit teaching and learning of NVCS is being increasingly implemented in training programs to prepare healthcare professionals [7, 10], we found here that views on explicit teaching of non-verbal skills were variable. This emphasizes a need for strategies, such as introducing a clear framework for explicit teaching, to improve student awareness of, and focus on, non-verbal communication skills [25]. It is important to note that, with or without explicit teaching, participants in this study perceived there to be an inherent capability to develop and ‘pick up’ on NVCS over time. However, this perception is confounded. Our participants were learning and teaching in a context where explicit teaching *was* included and *could* act as a foundation for the experiential, observational and feedback-based development of this knowledge. Our data cannot speak to whether these skills can be “picked up” without this explicit teaching of basic knowledge, or whether body language and facial expression can be effectively taught in any context. However, our data do indicate that a didactic approach is not challenged by a move online and can be preserved.

Experiential and observational strategies that promote active learning, such as role-play [26], were described by participants in this study as being an effective means to learn non-verbal communication skills. This is in line with reported perceptions of students in the literature [27, 28]. Further, our participants described picking up skills from other stakeholders in the simulated clinical scenarios, even when not participating in the roleplay themselves. This suggests the use of observational skills, as reported in the literature [29]; students model the behavior of other trusted participants, consciously or unconsciously, resulting in the development of NVCS over time [30]. In our data, the move to an online context challenged the students’ ability to observe and model tutor behaviors, but also highlighted another loss we had not theorized: the peer observation and modelling that takes place in a physical classroom.

The importance of peer interaction was also reflected in this study, whereby it was perceived that integrating feedback from multiple perspectives- the tutor, the simulated patient *and peers*- is useful in learning NVCS. Feedback allows learners to adjust their non-verbal communication skills with the intention to better meet the unique needs of each patient [25, 31, 32]. The tutors interviewed here described the importance of making students aware of what they are doing as part of their feedback approach. While feedback is frequently incorporated into learning workshops [1], it may be necessary to further study how integrating feedback from multiple perspectives influences skill development.

### Findings: How are These Learning Processes and Experiences Translated to an Online Environment?

Participants in this study reflected that in the rapid transition to online learning, teaching and learning of non-verbal communication was broadly preserved in the video-conferencing format, which is supported by perspectives of school-level educators reported previously [3]. However, many participants highlighted framing of individuals on the Zoom videoconferencing platform to be a central barrier to experiential and observational learning and feedback, which, to our knowledge, has not been reported previously. In this context, participants described the use and perception of non-verbal signals such as proximity and body language to be limited. For instance, in-person patients associate *leaning in* with a greater sense of connection [25]. In our data, however, the online experience of *leaning in* was described by a simulated patient as threatening. Similarly, previous studies have highlighted student uncertainty around where to look in a virtual environment [34], which was supported here by participants who raised the importance of adjusting eye contact for an online setting.

In the online setting, participants experienced limited peer feedback, and multi-source feedback from all stakeholders was described as incomplete, with a shift in focus to verbal communication. This has also been described by examiners, who find it difficult to evaluate NVCS online [13]. Conversely, a potential benefit to the Zoom platform raised by our interviewees was the ability to improve self-assessment and awareness from the self-view video. This is supported by findings that suggest Zoom heightened awareness of nonverbal behaviors, and video recordings allowed retrospective self-assessment [33]. With this considered, virtual platforms with video are becoming increasingly common and show some potential in improving self-reflection and performance of NVCS [31, 35]. However, few of these studies have explored whether improved NVCS online translates to a face-to-face setting. Knowing that non-verbal communication is differently interpreted based on the clinical environment, it is important that students are trained in both contexts. Noting that healthcare is increasingly virtual, and telehealth has become more common, future clinicians will need to be fluent in both online and face-to-face environments and be prepared to deploy their NVCS in both.

### Limitations

While this study is one of the first to gather a multi-perspective view on learning NVCS, there are several limitations to the scope and, therefore, interpretations of this study. Perhaps most obviously, there is a practical limitation in our sampling. We synthesized fewer student perspectives than perspectives from tutors or simulated patients, and these students were also limited in their ability to reflect on “business as usual” prior to the online conversion, given their lesser experience with face-to-face teaching. While this was largely a practical issue (students eligible for participation were already attempting to adapt to studying under unprecedented global circumstances and were not pressed for participation), it does limit the degree to which we can draw inferences about the student experience of the processes described by tutors and simulated patients. Conversely, we would note that the student perspective is often foregrounded in these types of analysis and there are already published papers (e.g. Dewi et al. [19]) that would allow for further exploration of these views. Simulated patients, by comparison, are only rarely included in research and we therefore offer underrepresented and novel perspectives to this discussion, despite the small number of students.

There are also three more conceptual limitations to consider. First, this study was focused on learning processes rather than outcomes. To this point, we solely collected interview data on perceptions of skill development. Quantitative outcome data (e.g. test scores) could be collected in future studies to explore how these processes relate to performance and applications in the clinical context. Secondly, for the purposes of standardizing the experience and allowing the individual experiences to be the main driver of variation, we collected data in only one graduate-entry medical program. Future research should build on this data by considering other graduate programs, undergraduate medical programs, as well as whether the findings generalize to the learning of NVCS in other clinical fields (nursing, dentistry, psychology). Finally, not all participants in the study have the benefit of hindsight to understand how non-verbal skills are important to the clinical environment. Accordingly, future research should incorporate the perspectives of stakeholders involved in different clinical settings and later phases of training to ensure a diversity of understanding.

## Conclusion

The current study describes the perspectives of tutors, simulated patients and medical students, offering a holistic view of the challenges and potential strategies for transitioning between face-to-face and online learning environments. Overall, we found that the perspectives of participants align with the prevailing literature on how communication skills can be taught and learned. Participants outlined four different learning modalities that, together, they perceive to be important for learning NVCS. Taken together, we suggest that, based on participant perceptions, learning NVCS online can be beneficial as the limited visual framing (often shoulders-up) of video conferencing will prepare students for telehealth clinical contexts. However, without a better understanding of how online and face-to-face environments translate, online education should be paired with face-to-face lessons. As the online learning landscape continues to evolve, these insights can guide the development of innovative and engaging methods for teaching and learning of NVCS in medical education.

## Supplementary Information

Below is the link to the electronic supplementary material.

Supplementary file1 (DOCX 21 KB)

## Data Availability

Owing to the type of data collected, the datasets are not available for public access to minimize the risk of reidentification. Access to the transcripts is via reasonable request to corresponding author.
