# Supplementary material for: Perspectives on the Teaching and Learning of Non-Verbal Communication Skills: Tutor, Simulated Patient and Student Experiences of a Rapid Transition to Online Learning
Source: Med Sci Educ. 2026 Feb 13;36(2):875–84. doi: 10.1007/s40670-026-02666-y (PMC13197556; doi:10.1007/s40670-026-02666-y)
Supplement: Supplementary file 1 — Supplementary file1 (DOCX 21 KB) [file 40670_2026_2666_MOESM1_ESM.docx]

**Non-Verbal Clinical Skills Interview Protocol (2020/527)**

***Purpose:*** Focusing on part three of the Med. Ed. during Covid project: Multi-perspective view on teaching non-verbal clinical skills within a history taking context both in the classroom and online: interviews with students, clinical skills tutors and simulated patient actors on a) how they teach and model the non-verbal aspects of the clinical interaction, and, b) how the move to online has impacted this.

***Required Participants:*** a minimum of four of each of the three participant groups: MChD Phase 1 (first and second year) students, staff members and simulated patients, ideally including an additional four students to reach four per year group. Interview participants will be recruited until sufficient cross section of gender, age, cultural and medical training background has been achieved.

***Interview Approach:*** Semi-structured interviews will explore the teaching and modeling of non-verbal interpersonal clinical skills training for Phase 1 (first and second year students). Questions will be guided by Vogel et al (2018) non-verbal skills domains developed specifically for a history taking context within an undergraduate medical degree, and the CARE Measure (Mercer, 2004). The semi-structured approach will leave room for the interviewer to adapt to the emerging data.

***Length:*** Maximum 1 hour

***Mode:*** Online, via video call using the Zoom platform, with a unique meeting code and password protection for each interview, and audio recorded only via an external audio recording device for transcription and analysis.

**Interview Guide**

***Part 1: Introduction and background***

- What is your association with the [institution] Medical School?
- Please briefly describe any previous clinical experience
- Please briefly describe your association with clinical skills training

***Part 2: Non-Verbal Communication Skills in Clinical Settings***

- Please describe your experience with training in non-verbal communication and interpersonal skills (clinical or otherwise)
- From your perspective, what is the role of non-verbal communication in clinical settings?
- What are the physical signs of communication that might be used in a clinical setting during history taking? Discuss specific examples (e.g. body posture, facial expressions, eye contact, tone of voice)
- In a history taking context, what are the important aspects of the clinical interaction that might not be included in a key steps guide? Discuss specific examples (e.g. making the patient feel at ease, really listening, showing care and compassion)
- What is the impact of non-verbal communication on the clinical interaction?

***Part 3: Non-Verbal Communication Skills Pedagogy, and impact of COVID-19 and online learning***

- How can non-verbal skills be taught and/or modelled? How has this been taught and/or modelled in your experience?
- How has COVID-19 and the switch to fully online learning impacted upon the teaching and modeling of non-verbal skills?
- Have you any suggestions for how non-verbal skills training might be improved, particularly in online delivery?
- Any other comments?
